# Rhubarb-Derived Extracellular Vesicles Mitigate Oxidative Stress and Metabolic Dysfunction in an Alzheimer’s Cellular Model

**DOI:** 10.3390/nu17233771

**Published:** 2025-11-30

**Authors:** Eleonora Calzoni, Gaia Cusumano, Agnese Bertoldi, Husam B. R. Alabed, Roberto Maria Pellegrino, Sandra Buratta, Lorena Urbanelli, Gokhan Zengin, Carla Emiliani

**Affiliations:** 1 Department of Chemistry, Biology and Biotechnology, University of Perugia, 06100 Perugia, Italy; eleonora.calzoni@unipg.it (E.C.); gaia.cusumano@dottorandi.unipg.it (G.C.); agnese.bertoldi@dottorandi.unipg.it (A.B.); husambr.alabed@unipg.it (H.B.R.A.); roberto.pellegrino@unipg.it (R.M.P.); sandra.buratta@unipg.it (S.B.); lorena.urbanelli@unipg.it (L.U.); 2 Centro di Digitalizzazione del Patrimonio Culturale (CeDiPa), University of Perugia, 06123 Perugia, Italy; 3 Department of Biology, Science Faculty, Selcuk University, Konya 42130, Turkey; gokhanzengin@selcuk.edu.tr; 4 Centro di Eccellenza Materiali Innovativi Nanostrutturati (CEMIN), University of Perugia, Via del Giochetto, 06123 Perugia, Italy

**Keywords:** plant-derived extracellular vesicles (PDEVs), oxidative stress, reactive oxygen species (ROS), Alzheimer’s disease (AD), antioxidant activity, drug delivery

## Abstract

**Background/Objectives**: Extracellular vesicles derived from edible plants have emerged as bioactive nanostructures with potential therapeutic and nutraceutical properties and are currently being investigated as natural carriers for the treatment of oxidative stress-induced damage and oxidative stress-related diseases, including neurodegenerative disorders such as Alzheimer’s disease (AD). Recent studies suggest that PDEVs exhibit high stability within the gastrointestinal tract and selective tissue-targeting abilities, facilitating the efficient delivery of bioactive molecules. **Methods:** This study investigates the antioxidant effects of Rheum rhabarbarum-derived EVs by assessing the antioxidant activity through different in vitro assays and their effects on oxidative stress and energy metabolism in the cellular model of Alzheimer’s disease. **Results:** Rhubarb-derived EVs showed measurable antioxidant capacity in chemical assays and were non-toxic under the tested conditions. Treatment reduced intracellular ROS levels and modulated oxidative stress-related proteins, suggesting a potential protective effect against oxidative damage. Moreover, metabolic analysis revealed a decrease in glycolytic activity, indicating a potential restoration of cellular bioenergetic homeostasis. **Conclusions:** These results provide preliminary evidence supporting the nutraceutical interest of rhubarb-derived EVs in counteracting oxidative stress, while further studies will be needed to confirm their biological relevance and therapeutic potential.

## 1. Introduction

Chronic diseases represent a growing challenge for public health globally, stimulating the search for new therapeutic strategies [1]. In this context, natural products, especially bioactive compounds of plant origin, have attracted considerable attention for their potential in the treatment of several complex diseases. The genus *Rheum* (Polygonaceae) includes about 60 species of perennial herbaceous plants, widely used for their therapeutic properties [2,3,4]. Recent studies have confirmed the value of these plants, revealing the presence of compounds with interesting biological activities, including stilbenoids, tannins, and anthraquinones [2,3,5]. The most studied species are *R. rhaponticum*, *R. rhabarbarum*, and *R. lhasaense*, characterized by a rich phytochemical composition and a wide range of biological activities. *R.rhaponticum* and *R. rhabarbarum* are known for their abundance in stilbenes, anthraquinones, and flavonoids, compounds that confer antioxidant, anti-inflammatory, and antimicrobial properties to these plants [6,7,8,9]. It has been demonstrated that *R. rhaponticum* extract exerts cardiovascular protection and modulates bone metabolism, with positive effects on bone mineral density and regulation of the estrogenic signaling pathway, while both *R. rhaponticum* and *R. rhabarbarum* extracts also exhibit significant anti-inflammatory and antioxidant activities [7,10,11]. Current research focuses on the identification of novel compounds, the characterization of their biochemical properties, and their potential application in the treatment of chronic diseases. In this context, plant-derived extracellular vesicles (PDEVs) have shown significant attention in recent years. These nanometric membranous particles (50–500 nm) play different roles within plant cells [12,13]. Although their mechanisms of action are not fully understood, their most well-documented role is in protecting plants against pathogens such as fungi, bacteria, and viruses [14,15]. One of the key characteristics of these vesicles is their stability, which allows them to protect biochemical components from environmental degradation. This property facilitates the transfer of molecular signals between different organisms, supporting intercellular communication among plant cells, animals, and microorganisms [16]. In fact, although plant-derived phytochemicals can be effectively extracted, many natural compounds tend to degrade or be metabolized into inactive derivatives in the circulatory system [17]. Beyond their role in plant defenses, PDEVs are naturally present in edible plant tissues and, consequently, are introduced into the human body through the diet [18,19,20]. For this reason, it has been hypothesized that they may contain bioactive biomolecules that reflect the characteristics of the plant cells of origin [13,21]. Recent studies have demonstrated that PDEVs exhibit high stability in the gastrointestinal tract and are efficiently internalized by mammalian cells, protecting encapsulated biomolecules from degradation and enabling their biological activity [22]. Upon reaching the target site, these vesicles can be internalized through endocytic pathways, facilitating the controlled release of their cargo and modulating key biological processes with antioxidant, anti-inflammatory, and antitumor effects [19]. In contrast to plant extracts, which, despite their high content of bioactive molecules, also have a complex potentially cytotoxic matrix that limits their bioavailability, PDEVs represent a more effective delivery system, ensuring greater selectivity and stability of the bioactive molecules [21,23]. This aspect has led to a growing interest in their use as natural food supplements, both for their intrinsic properties and as vehicles for the administration of bioactive compounds added after their isolation. Amid the growing interest in PDEVs, there is also their potential relevance to neurodegenerative diseases such as Alzheimer’s disease (AD). AD is characterized by progressive neuronal dysfunction accompanied by chronic neuroinflammation and higher oxidative stress, which contribute to synaptic loss and cognitive decline [24,25]. Oxidative damage to lipids, proteins, and nucleic acids is among the earliest detectable pathological events in AD, while prolonged activation of microglia and astrocytes perpetuates inflammatory cascades that exacerbate neuronal damage. Natural compounds with antioxidant and anti-inflammatory properties have therefore attracted considerable attention for their potential neuroprotective effects, and several plant-derived molecules, such as polyphenols, stilbenes, and flavonoids, have shown beneficial effects in cellular and animal models of AD [26,27,28]. Given that Rheum species are rich in antioxidant and anti-inflammatory phytochemicals, and that PDEVs can protect and deliver these molecules in a stable and bioavailable form, rhubarb-derived EVs may represent a promising candidate for counteracting oxidative stress and inflammatory pathways relevant to AD pathology.

This study aimed to isolate and characterize PDEVs from the rhizome of *R. rhabarbarum* and assess their antioxidant potential through in vitro assays and a fibroblast model of Alzheimer’s disease (AD), which exhibits systemic oxidative stress alterations relevant to AD. By combining biochemical and molecular analyses, it was possible to explore their concentration, morphology, and functional activity, and to offer new insights into their therapeutic potential as bioactive compounds.

## 2. Materials and Methods

### 2.1. Materials

Rhizoma of *Rheum rhabarbarum* L. was purchased from the local nursery. 2-(N-Morpholino)Ethanesulfonic Acid (MES), calcium chloride (CaCl_2_), sodium chloride (NaCl), methanol, and ethanol were purchased from Sigma Aldrich (St. Louis, MO, USA). 3-(4,5-dimethylthiazol-2-yl)-2,5-diphenyltetrazolium bromide (MTT), 2′,7′-Dichlorodihydrofluorescein diacetate, Phosphate-Buffered Saline (PBS), Dimethyl Sulfoxide (DMSO), glutaraldehyde (25%), 2,2-Diphenyl-1-picrylhydrazyl (DPPH), ABTS radical cation (2,2′-azino-bis(3-ethylbenzothiazoline)-6-sulphonic acid), TPTZ (2,4,6-tri(2-pyridyl)-s-triazine), FeCl_3_, Cu(II), neucoproine and Trolox were purchased from Sigma-Aldrich. Human dermal fibroblasts (HDFs) were obtained from the waste of skin biopsies of both healthy donors (HDF CTRL) and patients diagnosed with Alzheimer’s disease, classified as Clinical Dementia Rating scale 2 (HDF AD CDR2). In both cases, the isolation of HDF cells occurred occasionally, and all procedures were conducted with the informed consent of donors, adhering to the principles of the Helsinki Declaration [29]. The study was approved by the University of Perugia Ethics Committee with approval number Prot. N. CE-2492/25 (7/23/2025). The cells were cultured under standard conditions. Dulbecco’s modified Eagle’s medium (DMEM), fetal bovine serum (FBS), trypsin, and penicillin/streptomycin were purchased from Euroclone (Pero, Italy). All the other chemicals were of analytical grade and were obtained from Sigma-Aldrich unless otherwise indicated. Seahorse XF Glycolytic Rate Assay Kit, XF DMEM, XF glutamine, XF pyruvate, and XF glucose were purchased from Agilent (Agilent, Santa Clara, CA, USA).

### 2.2. PDEVs from Rheum Rhabarbarum Purification Method

For the isolation of EVs from Rhubarb rhizome, 2.5 g of dried material was weighed and resuspended in 10 mL of VIB buffer (20 mM MES, 2 mM CaCl_2_, 0.1 M NaCl, pH 6.0) and left under agitation overnight at 4 °C to allow rehydration. EVs’ enriched fraction isolation was performed using differential ultracentrifugation (dUC) [30]. First, centrifugation was carried out at 700× *g* for 20 min at 4 °C, and then the recovered liquid was filtered through a 0.45 µm membrane to remove possible debris. Subsequently, filtered samples underwent sequential centrifugation at increasing speeds (10,000× *g* for 60 min and 100,000× *g* for 60 min). The final pellet was then resuspended in 200 µL of PBS with 10% DMSO for biological assays, 100 µL of ethanol to test antioxidant activity, and 200 µL of methanol for QTOF LC/MS analysis. Moreover, the isolation of EVs was also performed by size-exclusion chromatography (SEC). EVs isolated using dUC and suspended in 200 µL of PBS were loaded on the ready-made columns IZON 70 nm (IZON Science, Cambridge, MA, USA). A total of 12 fractions (a 500 µL volume for each) were collected by using the extraction buffer for elution. Protein quantity in each fraction was measured using the Bradford assay [31]. EVs isolated by SEC were then analyzed in terms of concentration and morphology by NTA.

### 2.3. Nanoparticle Tracking Analysis (NTA) and Scanning Electron Microscopy (SEM)

The EV concentration and size distribution were analyzed using Malvern Panalytical NanoSight NS300 (NTA) (Malvern, Westborough, MA, USA). Rhubarb rhizome EVs were diluted in filtered PBS and analyzed with five measurements per sample in two independent experiments, as previously described [21]. For SEM, EVs were resuspended in 50 µL of PBS Buffer (0.22 µm filtered), and protein quantification was performed using the Bradford method [31]. A quantity corresponding to 2 µg of proteins was diluted in 2 mL of 2.5% glutaraldehyde (*v*/*v* in PBS 1X) and fixed for 15 min. Subsequently, the samples were diluted in 15 mL of dd-water (0.22 µm filtered), placed in Vivaspin tubes (300 KDa cutoff), and centrifuged (3000× *g*, 3 min). After removing the eluate, EVs were washed twice with 10 mL of water and centrifuged under the same conditions. Two dilutions (1:500 and 1:1000) were prepared, and 20 µL of each was seeded onto 12 mm diameter glass coverslips and successively fixed and coated with a thin conductive layer for electron microscopy.

### 2.4. Dynamic Light Scattering (DLS)

The zeta potential of *Rheum rhabarbarum* EVs was measured using a Nicomp^®^ Nano N3000 Dynamic Light Scattering (DLS) system (Entegris, Billerica, MA, USA). This instrument was employed to determine the surface charge of the nanoparticles through zeta potential analysis. The system is equipped with a high-power red laser diode (15–100 mW, 630 nm) and two detectors, PMT and APD, positioned at 90 degrees. The applicable electric field can be adjusted from 1 to 250 V/cm. The Nicomp algorithm also allows for the resolution of close multi-modal distributions with high resolution.

### 2.5. Immunoblotting Analysis

The presence of plant vesicular marker TET8 in rhubarb-derived EVs was evaluated by immunoblotting analysis. Here, 10 μg of total protein of EVs or *R. rhabarbarum* crude extract were mixed with 5× sample buffer (1 M Tris-HCl, pH 6.8, 5% SDS, 6% glycerol, 0.01% bromophenol blue) supplemented with 125 mM DTT. Samples were heated at 95 °C for 5 min and subjected to SDS-PAGE on 10% acrylamide gels. Gels were transferred onto polyvinylidene difluoride (PVDF) membranes using the Trans-Blot Turbo Transfer System (Bio-Rad, Hercules, CA, USA). After blocking, membranes were incubated overnight at 4 °C with a primary antibody against TET8 from PhytoAB (San Jose, CA, USA) and Calnexin from Santa Cruz Biotechnology (Santa Cruz, CA, USA). Detection was performed using HRP-conjugated secondary antibodies (Cell Signaling Technology, Beverly, MA, USA) and visualized via enhanced chemiluminescence (ECL) (GE Biosciences, Piscataway, NJ, USA).

### 2.6. In Vitro Antioxidant Capacity

The DPPH (2,2-diphenyl-1-picrylhydrazyl) radical scavenging capacity of *Rheum rhabarbarum* rhizoma EVs was assessed through the Blois method with few modifications [32]. Briefly, 50 μL of EVs was combined with 150 μL of freshly prepared DPPH solution. The mixture was then vigorously shaken and incubated in the dark at room temperature for 30 min. After incubation, the absorbance was recorded at 517 nm with an Infinite Tecan Spectrophotometer (Tecan Group Ltd., Mannedorf, Switzerland). The ABTS^+^ free radical scavenging activity was assessed following the method described by Miller et al. (1993) [33]. The ABTS^+^ radical was generated through the oxidation of ABTS with potassium persulfate. The stock solution of ABTS^+^ was then diluted in methanol to achieve an absorbance of 0.700 ± 0.020 at 734 nm. Also in this case, 50 μL of EVs was mixed with 150 μL of the diluted ABTS^+^ solution, and the absorbance was recorded at 734 nm after 6 min of incubation in the dark at room temperature.

In both cases, the percentage of DPPH and ABTS radical scavenging was calculated using the formula [34]:

$$\%\;\mathrm{Scavenging}\;\mathrm{effect}=\frac{\left(A0-A1\right)}{A0}\times 100$$

where *A*0 represents the absorbance of the control, and *A*1 corresponds to the absorbance of the sample.

The antioxidant capacity was also assessed using the FRAP (Ferric-Reducing Antioxidant Power) and CUPRAC (Cupric-Reducing Antioxidant Capacity) assays, following the previously described protocol [35]. For the FRAP assay [36], the reagent was prepared by mixing 300 mM acetate buffer, 10 mM TPTZ (2,4,6-tri(2-pyridyl)-s-triazine) in 40 mM HCl, and 20 mM FeCl_3_ in a volume ratio of 10:1:1. Then, 10 µL of the sample was mixed with 190 µL of FRAP reagent, and the mixture was incubated in the dark for 30 min at room temperature. Absorbance was measured at 593 nm using the Infinite Tecan instrument (Tecan Group Ltd., Mannedorf, Switzerland), and results were expressed as micromol of Trolox equivalents (TE) per 10^6^ particles per mL. If the absorbance exceeded the linear range of the Trolox standard curve, the sample was appropriately diluted. For the CUPRAC assay, the reagent was prepared by mixing 10 mM Cu(II), 7.5 mM alcoholic neocuproine solution, and 1 M acetate buffer (pH 7) in a volume ratio of 1:1:1. A total of 30 µL of the sample or Trolox standard solution was added to 170 µL of CUPRAC reagent and incubated for 30 min in the dark. Absorbance was measured at 450 nm using the Infinite Tecan reader (Tecan Group Ltd., Mannedorf, Switzerland). Also, in this case, results were expressed as micromol of Trolox equivalents (TE) per 10^6^ particles per mL.

### 2.7. Determination of Total Phenolic Content (TPC)

The total phenolic content of rhubarb-derived EVs was determined using the Folin–Ciocalteu assay [37]. A standard calibration curve was prepared using gallic acid as the reference compound. A volume of 50 µL of rhubarb-derived EVs was mixed with 150 µL of Folin–Ciocalteu reagent, which was previously diluted 10-fold with deionized water. The mixture was thoroughly vortexed and incubated for 5 min at room temperature. Subsequently, 150 µL of 20% Na_2_CO_3_ solution was added, and the reaction mixture was kept in the dark for 30 min at room temperature. The absorbance was measured at 750 nm using an Infinite Tecan Spectrophotometer (Tecan Group Ltd., Mannedorf, Switzerland). The results were expressed as micrograms of gallic acid equivalents per 10^6^ particles.

### 2.8. Untargeted Analysis of Polyphenol Content by Q-TOF LC/MS Mass Spectrometry

The extraction of polyphenols from rhubarb-derived EVs was performed by adding 40 µL of the extraction solution (70% methanol with 3% formic acid) to EV samples. After extraction solution addition, the samples were vortexed for 10 min using a thermomixer (Eppendorf, Hamburg, Germany) and centrifuged at 10,000× *g* for 10 min at 4 C. The resulting supernatant was collected and transferred into glass vials for subsequent injection into the mass spectrometer. Untargeted polyphenol profiling was performed using an Agilent 1260 Infinity II liquid chromatograph (Agilent, Santa Clara, CA, USA) coupled with an Agilent 6530 Accurate-Mass Q-TOF (quadrupole time-of-flight) mass spectrometer equipped with an Agilent JetStream electrospray ionization (ESI) source. Chromatographic separation was carried out using a Waters Acquity XBridge BEH Amide (HILIC) C18 column (Waters Corporation, Milford, MA, USA) (150 mm × 2.1 mm, 2.51.7 µm) maintained at 25–30 °C, with a mobile phase flow rate of 0.35 mL/min. The mobile phase consisted of water (Solvent A) and acetonitrile (Solvent B), both containing 0.2% formic acid. The chromatographic conditions were optimized based on previously published methods [38]. The gradient used was as follows: a linear gradient from 0 min (B = 5%) to 15 min (B = 45%), followed by another linear gradient from 15 min (B = 45%) to 18 min (B = 95%). The composition was then held at B = 95% from 18 to 20 min, before returning to B = 5% at 20.1 min. The run stopped at 23 min. The mass spectrometer operated in both positive and negative ionization modes under the following conditions: ion spray voltage at 3500 V, gas temperature at 250 °C, sheath gas temperature at 300 °C, nebulizer pressure at 35 psi, and sheath gas flow at 12 L/min. Data-dependent acquisition was applied in the 40–1700 m/z range for both MS and MS/MS, with a collision energy of 30 V.

Raw data were processed for peak detection and compound annotation using Qualitative Analysis MassHunter software (version 4.9) [21] and MS-DIAL software (version 4.9). Since no internal standards were used, the relative concentration of each detected polyphenol was estimated based on its contribution to the total phenolic content (TPC) determined via spectrophotometry [37]. The TPC value was used as a reference to express the relative percentage abundance of individual polyphenol species. Three independent replicates were performed for each sample.

### 2.9. Cell Treatments

#### 2.9.1. Cell Culture

Human dermal fibroblasts (CTRL and AD) were used as an in vitro model to evaluate the antioxidant activity of *R. rhabarbarum* EVs. The isolation of human dermal fibroblasts occurred from waste materials from two patients who underwent surgical procedures, and all procedures were conducted adhering to the principles of the Helsinki Declaration. The HDF CTRL and HDF AD cell lines were cultured in a Dulbecco’s modified Eagle’s medium (DMEM) containing 10% (*v*/*v*) heat-inactivated FBS and penicillin 10,000 U per mL/streptomycin 10 mg per mL and incubated in a humidified atmosphere (5% CO_2_ at 37 °C).

#### 2.9.2. *R. rhabarbarum* EV Cellular Uptake

To evaluate the cellular uptake of rhubarb-derived EVs, HDF cells were incubated with EVs previously labeled with 1,1′-Dioctadecyl-3,3,3′,3′-Tetramethylindocarbocyanine Perchlorate (DiL; Thermo Fisher Scientific, Carlsbad, CA, USA) and analyzed by fluorescence microscopy. For vesicle labeling, 50 µM DiL was added to the supernatant obtained after centrifugation at 10,000× *g*, while PBS processed in parallel was used as a negative control. After 30 min of incubation at room temperature, the excess dye was removed by washing the EVs with PBS through ultracentrifugation at 100,000× *g* for 70 min at 4 °C. For the uptake assay, HDF cells (5 × 10^4^) were exposed to the DiL-labeled NVs for 1 h, fixed with 4% paraformaldehyde for 20 min, and stained with FITC-conjugated phalloidin for 30 min to visualize F-actin filaments. Nuclei were counterstained using VECTASHIELD^®^ Vibrance Antifade Mounting Medium (Sigma-Aldrich, St. Louis, MO, USA) containing DAPI. Fluorescence images were acquired with a Nikon Eclipse TE2000-S fluorescence microscope (Minato City, Japan) equipped with an F-View II FireWire camera (Olympus Soft Imaging Solutions) and analyzed using CellF Imaging Software (Olympus Soft Imaging Solutions) [39].

#### 2.9.3. MTT Cytotoxicity Assay

To assess the effect of *R. rhabarbarum* EVs on cell proliferation, the MTT assay was performed [21]. Specifically, 1 × 10^4^ HDF CTRL and HDF AD cells were seeded in 96-well clear flat-bottom microplates in 100 μL of DMEM culture medium. After 24 h, the medium was replaced with 100 μL of fresh medium containing different dilutions of EVs. Each dilution was tested in octuplicates, with one set of wells serving as a control (100 μL of DMEM) and additional octuplicates used to account for the effects of PBS + DMSO (vehicle controls) at the same concentrations as the tested samples. The following day, the MTT assay was carried out as previously described [21]. Cell viability was calculated as the optical density percentage of treated cells compared to vehicle controls, assuming the control absorbance as 100% (absorbance of treated wells/absorbance of control wells × 100) using a Tecan Infinite Spectrophotometer (Tecan Group Ltd., Mannedorf, Switzerland).

#### 2.9.4. Trypan Blue Exclusion Assay

Cell growth was also monitored using Trypan blue dye staining with an automated cell counter (Invitrogen™ Countess™, Thermo Fisher Scientific, Waltham, MA, USA). Specifically, 5 × 10^4^ HDF CTRL and HDF AD cells were seeded into Falcon^®^ 24-well clear flat-bottom plates (Becton, Dickinson and Company, Franklin Lakes, NJ, USA) in triplicate. After 24 h, the culture medium was replaced with 500 µL of fresh medium containing different dilutions of EVs. Cell growth was assessed at 24 h using a 0.04% Trypan blue solution (Sigma-Aldrich, St. Louis, MO, USA).

#### 2.9.5. Intracellular ROS Production Assay

Intracellular ROS levels were assessed using the H_2_DCFHDA assay, which detects DCF fluorescence generated in the presence of ROS, as previously described [21]. Briefly, 10 × 10^3^ HDF CTRL and HDF AD cells were seeded into a 96-well black round-bottom polystyrene microplate with 100 μL of DMEM culture medium. The following day, CTRL and HDF AD cells were exposed to hydrogen peroxide (H_2_O_2_) at 250 µM, with or without co-treatment with *Rheum rhabarbarum* EVs at a high concentration corresponding to ~1.1 × 10^6^ particles. A quadruplet was kept as vehicle control to take into account the effect of DMSO. After 24 h, the H_2_DCFHDA assay was performed, and the fluorescence intensity of oxidized DCF was measured. Data, expressed as the percentage of DCF fluorescence intensity relative to vehicle controls, were normalized to cell viability, which was evaluated using the MTT assay. For this, cells were seeded in parallel onto a 96-well clear flat-bottom microplate and subjected to the same treatments and conditions. All measurements were performed in quadruplicate across three independent experiments.

#### 2.9.6. Immunoblot Analysis in CTRL and AD Cells

To assess the antioxidant activity of *R. rhabarbarum* EVs, 1.5 × 10^5^ cells were seeded in 6-well clear flat-bottom microplates in 1000 μL of DMEM culture medium. The following day, the medium was replaced with 1000 μL of fresh medium containing EVs at high concentrations corresponding to 1.1 × 10^6^ particles. After treatment, cells were incubated for 24 h, and cell lysate (30 μg) was subjected to 10% SDS-PAGE under reducing conditions following Laemmli’s method [40]. Proteins were then transferred onto a polyvinylidene difluoride (PVDF) membrane, as previously described [21]. The PVDF membrane was incubated overnight at 4 °C for 12 h with primary antibodies targeting Catalase, SOD1, and COX IV, followed by detection using the ECL (enhanced chemiluminescence) system, and images were acquired with an iBright 1500 imager system. Densitometric analysis of immunoblot images was performed using ImageJ software (version 1.54p).

### 2.10. Seahorse Glycolytic Activity Analysis

To evaluate the effect of *R. rhabarbarum* EVs on the glycolytic activity of CTRL and AD cells, the Agilent Seahorse XFp Extracellular Flux Analyzer (Agilent, Santa Clara, CA, USA). was used following the Glycolytic Rate Assay protocol. Cells were seeded at a density of 2.5 × 10^4^ cells/well, previously optimized to ensure linear signal response and avoid oxygen diffusion limitations, in XFp Cell Culture Microplates (Agilent, Santa Clara, CA, USA)., and after 24 h, the medium was replaced with fresh medium for CTRL and AD cells, and with fresh medium containing *R. rhabarbarum* EVs at high concentrations (1.1 × 10^6^ particles/mL, applied for 24 h). The following day, the medium was replaced with phenol red-free XF DMEM supplemented with 10 mM glucose, 2 mM sodium pyruvate, and 2 mM glutamine. Mitochondrial respiration was inhibited by adding Rotenone (0.5 µM) and Antimycin A (0.5 µM) (Rot/AA), which block the electron transport chain and prevent CO_2_-derived proton production. Then, 2-deoxy-D-glucose (2-DG), a glucose analogue, was introduced to competitively inhibit hexokinase, the key enzyme initiating glycolysis, thereby suppressing glycolytic activity. The effects of these treatments were assessed by measuring the Proton Efflux Rate (PER), a key parameter for determining glycolytic activity resulting from cellular metabolism [41,42]. All Seahorse parameters were normalized to protein content per well, determined by the Bradford assay, and each experiment was performed with at least three technical replicates and three independent biological replicates. Data were analyzed using Seahorse Wave software.

### 2.11. Statistical Analysis

The data shown in this study are reported as the mean values of three independent experiments, and differences among the samples were assessed using an ANOVA test. All statistical tests were performed using GraphPad Prism 9.00.

## 3. Results

### 3.1. Rhubarb-Derived Extracellular Vesicle Characterization: NTA and SEM Analysis

**Figure 1:**
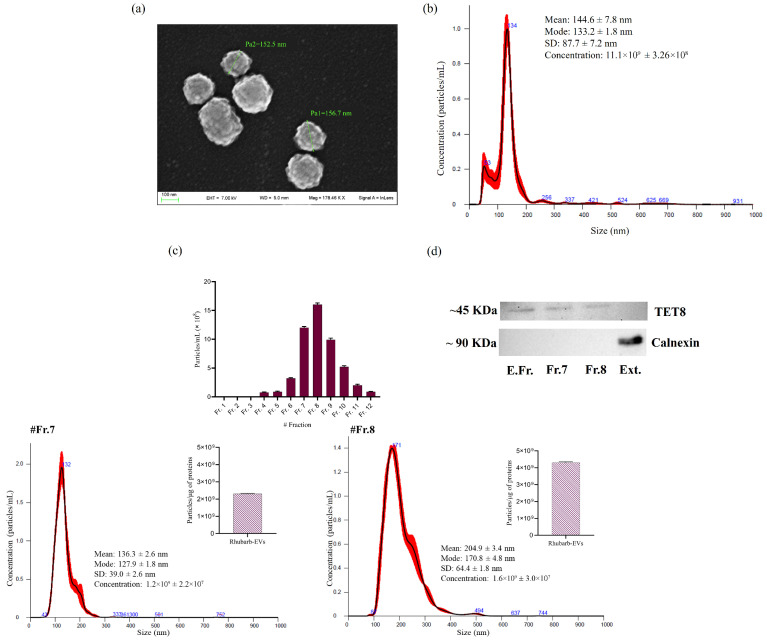
SEM images (**a**) and NTA (**b**) of the rhubarb-enriched fraction of EVs. Particle distribution obtained by NTA on EV SEC fractions and NTA of fractions 7 and 8 with the ratio of the particle number per μg of the protein that is indicative of the protein enrichment of each EV isolation by SEC (**c**), with TET8 and Calnexin detection in enriched fractions (E.Fr) of EVs and Fr. 7 and Fr. 8 obtained by SEC and *R. rhabarbarum* extract (Ext) obtained by immunoblotting (**d**). Data are expressed as mean ± SD (*n* = 3).

The presence of rhubarb-derived EVs in the enriched fractions determined through morphological analysis was first assessed by Scanning Electron Microscopy (SEM) (Figure 1a). SEM images revealed the presence of round-shaped vesicular structures with a heterogeneous size distribution, displaying a slightly textured surface. Nanoparticle tracking analysis (NTA) was then performed on the enriched EV fraction obtained after differential ultracentrifugation (Figure 1b). The results demonstrated that rhubarb EVs had a mean size of approximately 144.6 ± 7.8 nm, with a mode size of 133.2 ± 1.8 nm. Additionally, the total EV yield was quantified, reaching approximately 4.4 × 10^9^ particles/g of rhizome starting material. Moreover, the enriched rhubarb-derived EV zeta potential resulted in negative values (−14.65 mV) (Figure S1). These values are comparable to other PDEV zeta potentials and provide an indirect measure of EVs’ colloidal stability, which controls both vesicle–vesicle and vesicle–medium interactions.

To further improve purity, the enriched EV fraction was subjected to size-exclusion chromatography (SEC) using a 70 nm cutoff column. Twelve fractions were collected and analyzed by NTA (Figure S2). Fractions 7 and 8 exhibited the highest particle concentrations (1.2 × 10^9^ and 1.6 × 10^9^ particles/mL, respectively) and showed monodisperse size profiles centered at approximately 136 nm (Fr. 7) and 200 nm (Fr. 8) (Figure 1c). The ratio of the number of particles per μg of protein was determined as a quantification of the purity of EV preparations. This ratio was above 2 × 10^9^ for fraction t and 2 × 10^9^ for fraction 8, which, according to Webber and Clayton, corresponds to pure EV samples [43].

To validate the vesicular nature of the rhubarb-derived EVs, immunoblotting for the plant EV marker TET8 was performed on the enriched fraction and on SEC fractions 7 and 8 (Figure 1d). A clear positive signal for TET8 was detected in all EV-containing samples, corroborating the NTA results regarding vesicle enrichment and size homogeneity. In parallel, a crude extract from *Rheum rhabarbarum* was analyzed as a negative control. Calnexin, an endoplasmic reticulum protein commonly used as a non-EV marker, was detected only in the crude extract and not in the enriched or purified EV samples. This confirms the efficiency of the combined differential ultracentrifugation and SEC separation and the high purity of the isolated rhubarb-derived EVs.

Although plant-derived EVs are heterogeneous, both the enriched fraction obtained through differential ultracentrifugation and the SEC-purified fractions displayed a monodisperse size profile. The slight shift in particle diameter observed after SEC reflects the selective enrichment of specific EV subpopulations rather than increased variability, a phenomenon commonly reported for PDEVs. SEC also improved purity by removing protein aggregates and non-vesicular particles, yielding a stable and reproducible vesicle population suitable for functional analyses. These size and structural characteristics are consistent with previously described plant-derived EVs, including those from citrus, green tea, and aloe, which are known for their stability and ability to transport bioactive molecules [21,44,45].

### 3.2. In Vitro Antioxidant Capacity of Rhubarb-Derived Extracellular Vesicles

At the beginning, the antioxidant activity of rhubarb-derived EVs was analyzed through the DPPH (2,2-Diphenyl-1-picrylhydrazyl), ABTS (2,2′-Azino-bis(3-ethylbenzothiazoline-6-sulfonic acid) diammonium salt), CUPRAC (cupric-ion-reducing antioxidant capacity), and FRAP (ferric-reducing ability of plasma) assays, in order to determine their ability to neutralize free radicals and chelate metal ions as a first-level screening tool. Moreover, the IC50 was determined by linear interpolation from the dose–response curves, representing the concentration of rhubarb-derived EVs required to reduce free radical activity by 50% in the DPPH and ABTS assays or to achieve 50% of the maximal antioxidant response in the FRAP and CUPRAC assays. The obtained values were expressed in µg of EVs per 10^6^ particles, with standard deviations calculated from three independent experiments. Table 1 reports the results of the antioxidant capacity of rhubarb-derived EVs expressed in µmol of Trolox equivalents (TE).

The ABTS assay revealed an antioxidant capacity of 120.65 ± 6.75 µmol TE/10^6^ particles, while the DPPH showed a slightly lower value of 92.6 ± 11.45 µmol TE/10^6^ particles. These results indicate a broad-spectrum antioxidant activity, with a higher Trolox-equivalent antioxidant capacity detected using ABTS. On the other hand, the IC50 values, which represent the concentration required to inhibit 50% of radical activity, were 7.50 ± 0.26 µg/10^6^ particles for ABTS and 4.5 ± 0.09 µg/10^6^ particles for DPPH. The lower IC50 in the DPPH test indicates that the rhubarb-derived EVs contain highly potent radical scavenging compounds, which are effective in the interaction with DPPH radicals. The CUPRAC assay demonstrated an antioxidant capacity of 722.50 ± 2.30 µmol TE/10^6^ particles, whereas the FRAP assay yielded a lower value of 342.65 ± 6.95 µmol TE/10^6^ particles. These findings indicate that rhubarb-derived EVs exhibit important redox potential, with a greater overall electron-donating ability observed in the CUPRAC assay. In terms of IC50 values, the CUPRAC assay exhibited an IC50 of 250.80 ± 12.65 µg/10^6^ particles, while the FRAP assay displayed a significantly lower IC50 of 31.10 ± 2.85 µg/10^6^ particles. The lower IC50 in the FRAP assay suggests that the EVs harbor potent ferric-reducing constituents that efficiently participate in single-electron transfer reactions. In contrast, the higher IC50 in CUPRAC implies a weaker interaction with cupric ions, reflecting differences in redox potential and the mechanistic specificity of the antioxidant compounds present in rhubarb-derived EVs. These differences highlight the complexity of their antioxidant profiles and indicate the presence of heterogeneous bioactive molecules with different reduction potentials. Moreover, the magnitude of these Trolox-equivalent values is consistent with those reported for other antioxidant-rich plant-derived EVs. For example, citrus-derived EVs exhibited strong ABTS and DPPH scavenging activities with values of 369.11 ± 1.92 and 75.62 ± 0.77, respectively, which are consistent with our results [44]. Thus, these results place rhubarb-derived EVs within the range of antioxidant activities already documented for other PDEVs, supporting their classification as vesicles with a robust redox potential.

### 3.3. Untargeted Polyphenol Analysis Through LC/MS

To assess the presence of polyphenols in rhubarb-derived extracellular vesicles, an analysis was conducted using liquid chromatography coupled with mass spectrometry (LC-MS). Furthermore, to avoid the absence of co-contamination by polyphenols in the EV fraction, the spectrophotometric ratio A280/A230 was evaluated, obtaining a value of 1.92, which indicates a high purity of the preparation and avoids the significant presence of residual phenolic contaminants. Additionally, a semi-quantitative evaluation was performed, using the total polyphenol content (TPC) determined by the Folin method as a reference, as previously described. The analysis enabled the identification and quantification of a range of bioactive compounds, primarily polyphenols, as reported in Table 2.

Among the most abundant metabolites detected, (-)-epicatechin (4.73 µg/g), gallic acid (2.66 µg/g), and rhein (2.48 µg/g) were predominant. These polyphenols have previously been identified in extracts of rhubarb, suggesting that such secondary metabolites are actively transported through PDEVs [10]. Beyond these primary compounds, several flavonoids and proanthocyanidins with significant biological activity were also identified. Of note, procyanidin B1 (0.46 µg/g) and procyanidin B2 (0.10 µg/g) have demonstrated an antioxidant activity exceeding that of vitamin C [46]. Finally, among the identified phenolic compounds, trans-4-coumaric acid (0.20 µg/g) and naringenin (0.10 µg/g) are known modulators of antioxidant and inflammatory activity and have also been identified in other plant species [47,48]. These findings indicate that the cargo of rhubarb-derived EVs is characterized by many interesting secondary metabolites, such as polyphenols, which may have anti-inflammatory and antioxidant activity. This evidence is consistent with previous research highlighting the potential of EVs as bioactive compound carriers for therapeutic applications in neurodegenerative diseases.

### 3.4. Rhubarb-Derived Extracellular Vesicle Uptake

**Figure 2:**
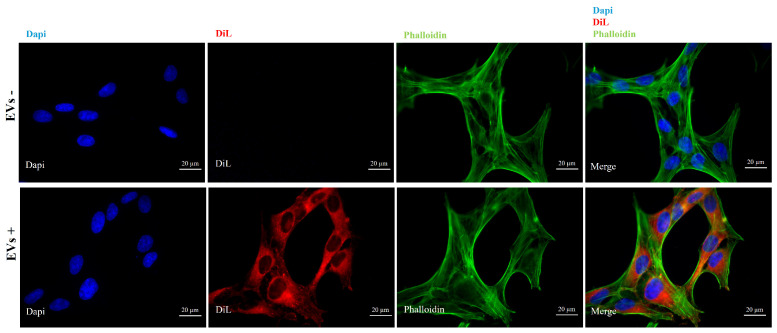
DiL-labeled EV internalization by HDF AD cells with the nuclei marker DAPI (blue, DAPI filter), along with relative phalloidin and merged images (image magnification: 60×). In the **upper panel**, HDF AD cells treated with the extraction buffer processed through the same differential centrifugation and DiL-labeling procedure are reported; in the **lower panel**, HDF AD cells incubated with DiL-labeled EVs (1.10 × 10^6^ particles) are reported.

To assess the intracellular uptake of *R. rhabarbarum* EVs, HDF cells were incubated with DiL-labeled vesicles and analyzed by fluorescence microscopy analysis, using dye-only controls processed through the same ultracentrifugation method to exclude non-specific labeling or dye aggregation (Figure 2).

Following exposure, a distinct red fluorescence signal was detected within the cytoplasmic region of the HDF AD cells, indicating that the rhubarb-derived vesicles were efficiently taken up by the cells rather than remaining attached to the cell surface. The fluorescence pattern appeared as punctate spots distributed throughout the cytoplasm, consistent with vesicular localization, and suggesting the accumulation of EVs within intracellular compartments. No fluorescence was detected in cells incubated with the extraction buffer processed through the same differential centrifugation and DiL-labeling procedure, confirming the specificity of the observed signal.

### 3.5. Intracellular Antioxidant Activity of Rhubarb-Derived EVs

**Figure 3:**
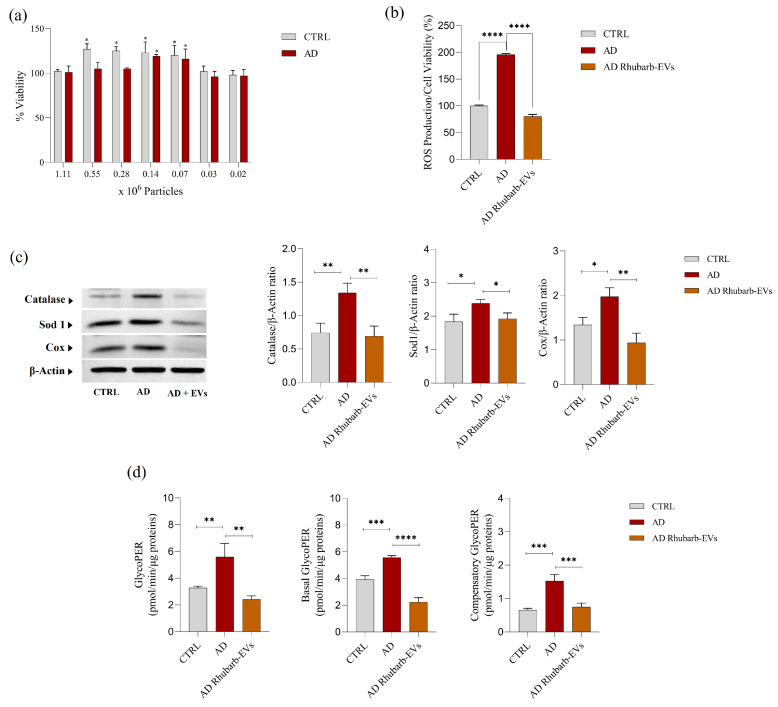
Effect of rhubarb-derived EVs on viability, oxidative stress, and glycolytic metabolism. (**a**) MTT cell viability assay in CTRL and AD cells with different concentrations of rhubarb-derived EVs (particles per million). (**b**) ROS levels in CTRL, AD cells, and AD cells treated with 1.10 × 10^6^ particles of rhubarb-derived EVs. (**c**) Representative Western blot images and densitometric analysis of oxidative stress-related proteins (Catalase, SOD1, and COX) in CTRL and AD cells, with and without EV treatment. (**d**) Seahorse Glycolytic Rate Assay results show GlycoPER, Basal GlycoPER, and Compensatory GlycoPER in CTRL, AD, and AD-treated cells. Data are expressed as mean ± SD; statistical significance is indicated as * *p* < 0.05, ** *p* < 0.01, *** *p* < 0.001, and **** *p* < 0.0001.

To investigate the biological activity of rhubarb-derived EVs after their uptake and the potential protective effect on AD cells, several studies were performed to assess the cytotoxicity, oxidative stress level, the expression of key oxidative stress-related protein markers, and cellular energy metabolism (Figure 3).

To assess the potential cytotoxicity of rhubarb-derived EVs, an MTT cell viability assay was performed on both control (CTRL) and AD cells treated with different EV concentrations, expressed as particles per million. As shown in Figure 3a, EV treatment did not compromise cell viability at any of the tested concentrations in both cell types. On the contrary, a slight increase in viability was observed, suggesting a potential stimulatory effect on cell growth at specific concentrations. These results were further confirmed by the Trypan blue exclusion assay, which showed that the tested concentrations of EVs were not cytotoxic for the analyzed cell lines (Figure S3). Moreover, in order to demonstrate the antioxidant effect of rhubarb-derived EVs, the basal levels of ROS produced by CTRL and AD cells and those produced by AD cells treated with high concentrations of EVs (1.10 × 10^6^ particles) were evaluated. In fact, one of the key pathological mechanisms in AD is increased oxidative stress, characterized by excessive production of ROS. As shown in Figure 3b, AD cells exhibited a significant increase in ROS levels compared to CTRL cells (*p* < 0.0001), indicating a pronounced oxidative imbalance. However, EV treatment resulted in a drastic reduction in ROS production and restoration to levels comparable to control cells. In addition, in order to exclude non-specific quenching by phenolic compounds present in the EVs, a cell-free control was performed in which EVs were incubated with the ROS-sensitive fluorescent probe in the absence of cells. No significant decrease in fluorescence was observed, indicating that the effect measured in cells reflects a true reduction in ROS. To further investigate the antioxidant effects of EVs, the expression levels of key oxidative stress response enzymes—catalase, SOD1, and COX—were analyzed (Figure 3c). AD cells exhibited an upregulation of SOD1 and catalase, compared to CTRL cells (*p* < 0.05 and *p* < 0.01, respectively), which is likely reflecting an adaptive response to oxidative damage. However, EV treatment reduced the expression of oxidative markers, suggesting a decrease in oxidative challenge rather than a direct modulation of antioxidant pathways, and indicating a reduced requirement for compensatory cellular defense mechanisms. Similarly, COX expression was elevated in AD cells, which may be associated with altered mitochondrial activity, whereas EV treatment led to a reduction in COX levels, consistent with a functional attenuation of mitochondrial stress. Finally, the impact of rhubarb-derived EVs on cellular energy metabolism was investigated through a Seahorse Glycolytic Rate Assay to assess total glycolytic activity (GlycoPER), basal glycolysis (Basal GlycoPER), and compensatory glycolysis (Compensatory GlycoPER) (Figure 3d). The GlycoPER value was calculated by excluding the mitochondrial contribution to the Proton Efflux Rate (PER), which was determined using Oxygen Consumption Rate (OCR) and Extracellular Acidification Rate (ECAR) measurements. The results indicate that AD cells exhibit a significant increase in GlycoPER compared to control cells (*p* < 0.01), suggesting a metabolic shift towards glycolysis as a compensatory mechanism for mitochondrial dysfunction. Both Basal GlycoPER and Compensatory GlycoPER also significantly increased, reflecting an overall intensification in glycolytic activity and an enhanced ability of AD cells to compensate for metabolic imbalances. Particularly, treatment with rhubarb-derived EVs led to a significant reduction in all analyzed parameters (*p* < 0.001), indicating a functional rebalancing of cellular energy metabolism and reduced dependence on glycolytic flux, without implying direct restoration of mitochondrial bioenergetics.

## 4. Discussion

Oxidative stress is one of the most widely studied cellular stressors, as it interferes with normal cellular functions and contributes to a variety of pathological conditions [49,50]. It is central to the onset and progression of many degenerative diseases, including neurodegenerative diseases (such as Alzheimer’s and Parkinson’s), cancer, and cardiovascular disease [50,51]. Given its widespread impact, the search for natural compounds with antioxidant properties has gained increasing attention as a potential strategy for disease prevention and therapy. In this study, the properties of EVs derived from edible *R. rhabarbarum* rhizome, focusing on their structural characteristics, antioxidant potential, and biological effects, have been explored in order to investigate their ability to modulate oxidative stress and energy metabolism in an in vitro model of neurodegeneration, providing new insights into their potential therapeutic applications.

Rhubarb-derived EVs were morphologically characterized using Scanning Electron Microscopy (SEM) and nanoparticle tracking analysis (NTA), revealing a population of vesicles with an average size of approximately 144.6 nm and a total yield of about 4.4 × 10^9^ particles per gram of rhizome. These size and structural characteristics are consistent with previously described plant-derived EVs, including those from citrus, green tea, and aloe, which are known for their stability and ability to transport bioactive molecules [21,44,45]. One of the most significant aspects of this study is the high antioxidant potential of rhubarb EVs, as demonstrated by in vitro assays (DPPH, ABTS, FRAP, and CUPRAC). Specifically, the EVs exhibited a strong free radical scavenging capacity, with IC50 values of 7.50 µg/10^6^ particles in the ABTS assay and 4.50 µg/10^6^ particles in the DPPH assay, indicating the presence of potent antioxidant compounds. These findings are comparable to those reported for polyphenol-rich extracellular vesicles from other plant species [52,53,54]. Moreover, the redox capacity detected in the CUPRAC and FRAP assays suggests that bioactive compounds contained within the EVs may exert protective effects through electron transfer mechanisms. The antioxidant values obtained are comparable to previously published data on plant-derived EVs [44,52]. These comparisons support the interpretation that the antioxidant profile of rhubarb-derived EVs aligns with the stronger antioxidant PDEVs described in the literature. In agreement with these biochemical properties, the metabolic profiling of rhubarb-derived EVs revealed the presence of a lot of interesting polyphenols, including (-)-epicatechin and gallic acid, which have previously been identified in extracts of *R. rhabarbarum* [10]. These secondary metabolites are well-documented for their potent antioxidant and anti-inflammatory activities, with evidence suggesting their neuroprotective effects in preclinical models of Alzheimer’s disease through mechanisms such as attenuation of oxidative damage, inhibition of amyloid-β aggregation, and modulation of signaling pathways involved in neuronal survival [55,56,57]. (-)-Epicatechin, a flavanol recognized for its neuroprotective and antioxidant properties, has been shown in preclinical studies to enhance endothelial function and mitigate oxidative damage in neurons, thereby modulating cellular stress [58,59]. Similarly, gallic acid, a phenolic acid with anti-inflammatory and neuroprotective effects, is capable of reducing reactive oxygen species (ROS) production and promoting neuronal survival in neurodegenerative disease models [60,61,62]. Rhein, an anthraquinone derivative, has been linked to beneficial effects on mitochondrial metabolism and autophagy regulation, with potential neuroprotective implications in conditions such as Alzheimer’s disease [63,64]. The detection of these polyphenols in rhubarb-derived EVs indicates their potential role as molecular cargo, supporting the hypothesis that plant-derived extracellular vesicles can act as natural nanocarriers for bioactive metabolites with therapeutic implications in neurodegenerative disorders. From a biological perspective, this study aims to evaluate the effects of EVs in a cellular model of AD. In particular, the present study employed human dermal fibroblasts derived from AD donors, a peripheral cellular model frequently used to investigate systemic alterations associated with AD. While fibroblasts cannot reproduce neuronal dysfunction, they exhibit mitochondrial impairment, increased ROS production, deficits in antioxidant systems, and metabolic alterations that mirror early systemic changes occurring in AD [65,66,67]. The MTT assay confirmed the absence of cytotoxic effects and the ability of rhubarb-derived EVs to reduce ROS levels in AD cells. Specifically, EV treatment significantly decreased ROS levels, restoring them to values comparable to those observed in control cells. This suggests a potential neuroprotective effect, likely mediated by bioactive molecules such as polyphenols and flavonoids, which are known for their antioxidant properties and have been reported in other plant-derived EV studies [21,52,68,69,70,71,72].

At the molecular level, the modulation of key antioxidant enzyme expression, specifically catalase and SOD1, further supports the role of rhubarb-derived EVs in oxidative stress regulation. The elevated levels of catalase and SOD1 in untreated AD cells indicate a compensatory response to chronic oxidative stress, a mechanism previously described in cellular and murine models of neurodegeneration [73]. However, EV treatment significantly reduced the expression of these enzymes, suggesting a normalization of intracellular redox balance and a reduced need for endogenous antioxidant defense activation. Another novel aspect of this study is the ability of rhubarb-derived EVs to modulate energy metabolism in AD cells. Glycolytic Rate analysis revealed a significant increase in glycolysis in AD cells compared to controls, indicative of a compensatory response to mitochondrial dysfunction, an extensively documented phenomenon in AD pathophysiology [74]. EV treatment led to a marked reduction in total glycolysis (GlycoPER), basal glycolysis (Basal GlycoPER), and compensatory glycolysis (Compensatory GlycoPER), indicating an association between EV exposure and metabolic rebalancing in this cellular model. This observation is particularly relevant given that bioenergetic dysfunction is considered an early event in AD progression and is increasingly recognized as a promising therapeutic target [75]. Overall, these findings suggest that rhubarb-derived EVs may act as modulators of oxidative stress and energy metabolism in AD cells, providing new insights into their potential therapeutic applications. In particular, their ability to reduce ROS levels and restore glycolytic balance strengthens the hypothesis of their neuroprotective properties. However, as these are preliminary in vitro studies, further in vivo investigations and cargo-specific analyses are required to confirm their efficacy and to elucidate their therapeutic potential.

## 5. Conclusions

Plant-derived extracellular vesicles have emerged as promising and versatile tools in biomedical research due to their ability to encapsulate, protect, and transport bioactive compounds that can interact with animal cells. The results of this study suggest that rhubarb-derived EVs may contribute to the regulation of oxidative stress and metabolic dysfunction, both of which are critical factors in the pathophysiology of Alzheimer’s disease. Moreover, these EVs are obtained from edible plant material, which highlights their potential as safe, naturally derived nutraceutical agents that could be directly incorporated into the diet or developed into functional food products. The consumption of rhubarb-derived EVs may represent a novel strategy to deliver bioactive compounds in a biocompatible and non-toxic form, overcoming some of the limitations associated with synthetic nanocarriers. Nonetheless, as these findings derive from preliminary in vitro experiments, further investigations are needed to confirm these effects and completely assess the therapeutic and nutraceutical potential of rhubarb-derived EVs.

## Figures and Tables

**Table 1:** Antioxidant of rhubarb-derived EVs. Results are expressed as µmol of Trolox equivalent per 10^6^ particles, and values are reported as means ± SD of three parallel measurements. Analyses were performed using Trolox as reference standards.

	µmol TE/10^6^ Particles	IC 50 (µg/10^6^ Particles)
ABTS	120.65 ± 6.75	7.50 ± 0.26
DPPH	92.6 ± 11.45	4.5 ± 0.09
FRAP	342.65 ± 6.95	31.10 ± 2.85
CUPRAC	722.50 ± 2.30	250.80 ± 12.65

**Table 2:** Identification of polyphenol species in rhubarb-derived EVs through LC/MS. The concentration is reported as µg of compound detected in samples obtained from 4.4 × 10^9^ particles, corresponding to the EV fraction isolated from 1 g of *R. rhabarbarum*.

Identified Metabolite in Rhubarb-Derived EVs	Concentration (µg/gr of Starting Material)
(-)-epicatechin	4.73 ± 1.89
Gallic acid	2.66 ± 1.06
rhein	2.48 ± 0.99
epicatechin gallate	0.52 ± 0.21
Procyanidin B1	0.46 ± 0.18
Procyanidin B2	0.10 ± 0.04
6-Cinnamoyl-1-galloylglucose	0.45 ± 0.18
Emodin	0.25 ± 0.10
trans-4-Coumaric acid	0.21 ± 0.08
Naringenin	0.11 ± 0.04
Salicylic acid	0.08 ± 0.03
Emodin 8-O-(beta)-D-glucoside	0.04 ± 0.02
Methyl gallate	0.04 ± 0.02
2-Hydroxyphenylacetic acid	0.03 ± 0.01
[3,4,5-trihydroxy-6-[[(E)-3-(4-hydroxyphenyl)prop-2-enoyl]oxymethyl]oxan-2-yl] 3,4,5-trihydroxybenzoate	0.03 ± 0.01
feruloyltyramine	0.03 ± 0.01
Eriodictyol-7-O-glucoside	0.02 ± 0.01
2,5-dihydroxy benzoic acid	0.02 ± 0.01
Lecanoric Acid	0.02 ± 0.01
Phloretin-2′-O-glucoside	0.02 ± 0.01
1,6-Digalloyl-beta-D-glucopyranose	0.01 ± 0.01
Biochanin-7-O-glucoside	0.01 ± 0.00
Homoeriodictyol	0.01 ± 0.00
Isoquercitrin	0.01 ± 0.00
isorhamnetin-3-rutinoside	0.01 ± 0.00
Kaempferol-3-O-glucoside-3″-rhamnoside	0.01 ± 0.00
p-Hydroxybenzaldehyde	0.01 ± 0.00
Quercitrin	0.01 ± 0.00

## Data Availability

The original contributions presented in this study are included in the article/Supplementary Materials. Further inquiries can be directed to the corresponding authors.

## Supplementary Materials

The following supporting information can be downloaded at https://www.mdpi.com/article/10.3390/nu17233771/s1, Figure S1. Analysis of Rheum rhabarbarum EVs by Dynamic Light Scattering (DLS) in order to evaluate the zeta potential. The zeta potential value was obtained after multiple analysis cycles for a total of 13 minutes of evaluation. Figure S2. Nanoparticle tracking analysis of Rheum rhabarbarum EV fractions obtained by SEC. Evaluation of particle size and concentration of different density gradient fractions (from 4 to 12) is reported as particles/mL obtained by NTA. Figure S3. Trypan blue exclusion assay on CTRL and AD cells treated with the same concentrations of EVs, tested with MTT assay, in order to evaluate the potential cytotoxicity. Data are expressed as mean ± SD of three replicates.

## Abbreviations

The following abbreviations are used in this manuscript:

AD	Alzheimer’s Disease
PDEVs	Plant-Derived Extracellular Vesicles
EVs	Extracellular Vesicles
SEM	Scanning Electron Microscopy
NTA	Nanoparticle Tracking Analysis
DPPH	2,2-Diphenyl-1-picrylhydrazyl
ABTS	2,2′-Azino-bis(3-ethylbenzothiazoline-6-sulfonic acid)
FRAP	Ferric-Reducing Antioxidant Power
CUPRAC	Cupric-Ion-Reducing Antioxidant Capacity
ROS	Reactive Oxygen Species
TEs	Trolox Equivalents
TPC	Total Polyphenol Content
SOD1	Superoxide Dismutase 1
COX	Cyclooxygenase
GlycoPER	Glycolytic Proton Efflux Rate
OCR	Oxygen Consumption Rate
ECAR	Extracellular Acidification Rate
