# Supplementary material for: Rhubarb-Derived Extracellular Vesicles Mitigate Oxidative Stress and Metabolic Dysfunction in an Alzheimer’s Cellular Model
Source: Nutrients. 2025 Nov 30;17(23):3771. doi: 10.3390/nu17233771 (PMC12694416; doi:10.3390/nu17233771)
Supplement: Supplementary file 1 [file nutrients-17-03771-s001.zip › nutrients-3990361-supplementary.pdf]

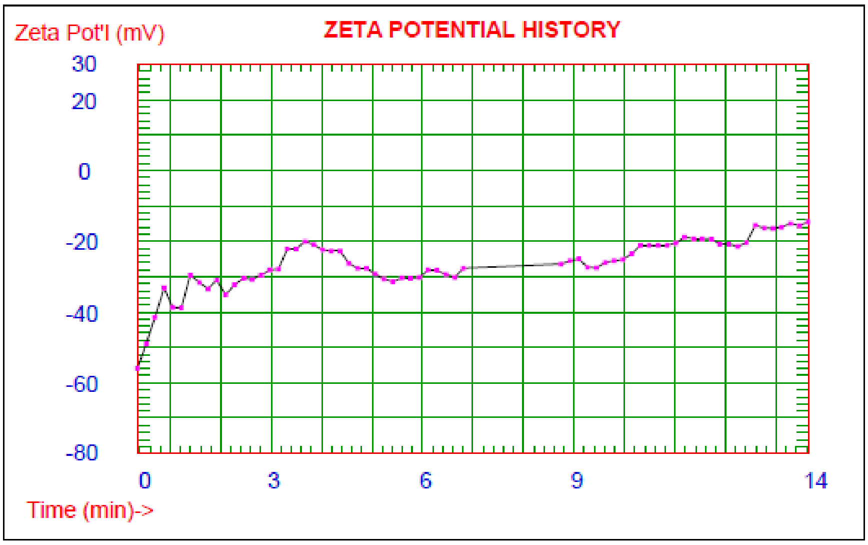

Sample Frequency:	-----
Reference Frequency:	-----
Cell Current:	14.13 mA (Cond.Factor = 0.70)
Avg. Phase Shift:	29.10 rad/sec.
Avg. Mobility:	-1.09 M.U.
Half-Width Mobility Dist.	-----
Avg. Zeta Potential:	-14.65 mV
Half-Width Zeta Pot'l Dist.	-----
Sample Temperature:	23 C
Liquid Viscosity:	0.933 cPoise
Index of Refraction:	1.333
Dielectric Constant:	78.500
Laser Wavelength:	635.0 nm
Scattering Angle:	-14.1 deg.
E-Field Strength:	6.00 V/CM
Channel Width:	20.0 uSec.
Run Time:	00:13:38

Figure S1. Analysis of *Rheum rhabarbarum EVs* by Dynamic Light Scattering (DLS) in order to evaluate the Zeta-potential. The zeta potential value was obtained after multiple analysis cycles for a total of 13 minutes of evaluation.

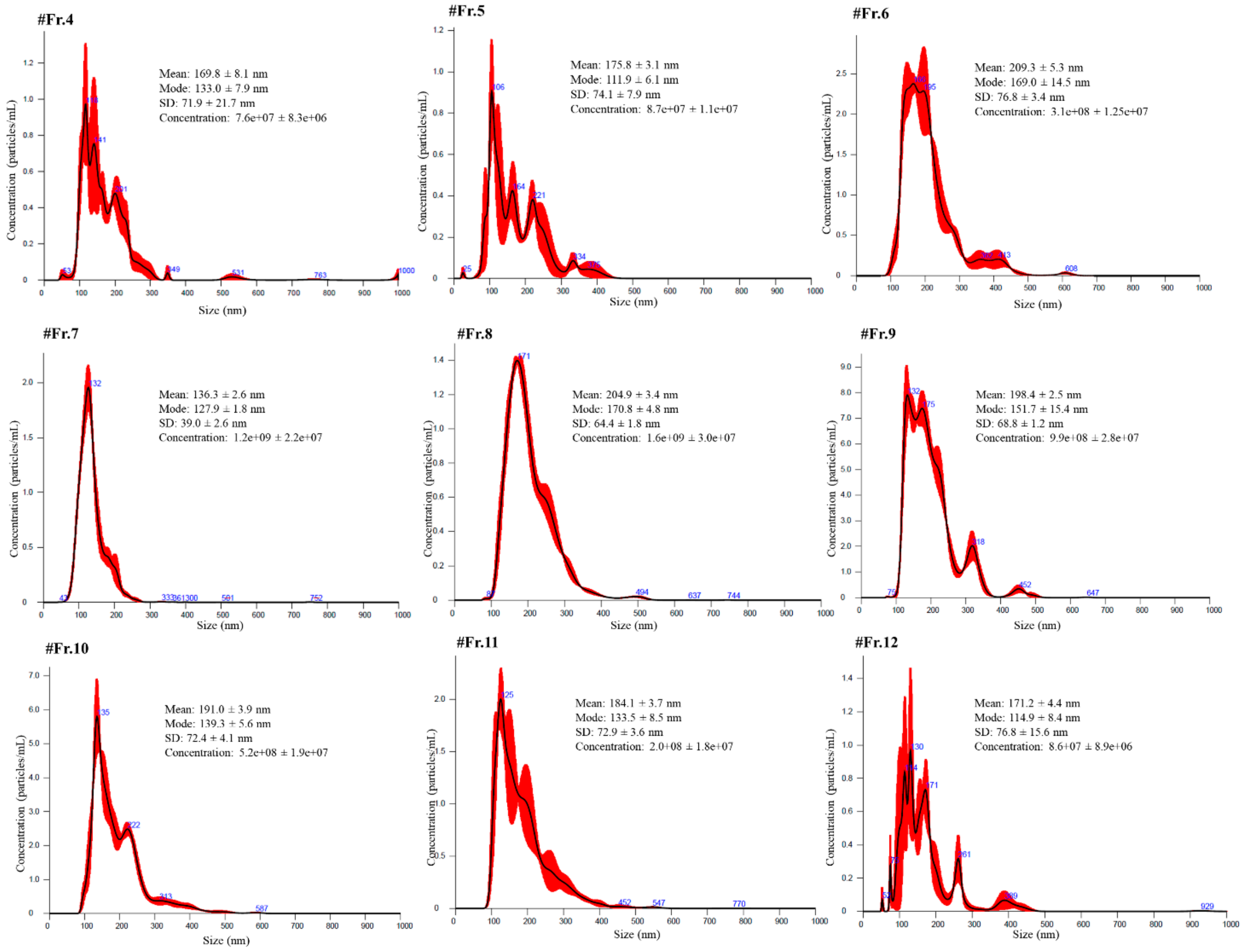

Figure S2. Nanoparticles Tracking Analysis of *Rheum rhabarbarum EVs* fractions obtained by SEC. Evaluation of particle size and concentration of different density gradient fractions (from 4 to 12) is reported as Particles/mL obtained by NTA.

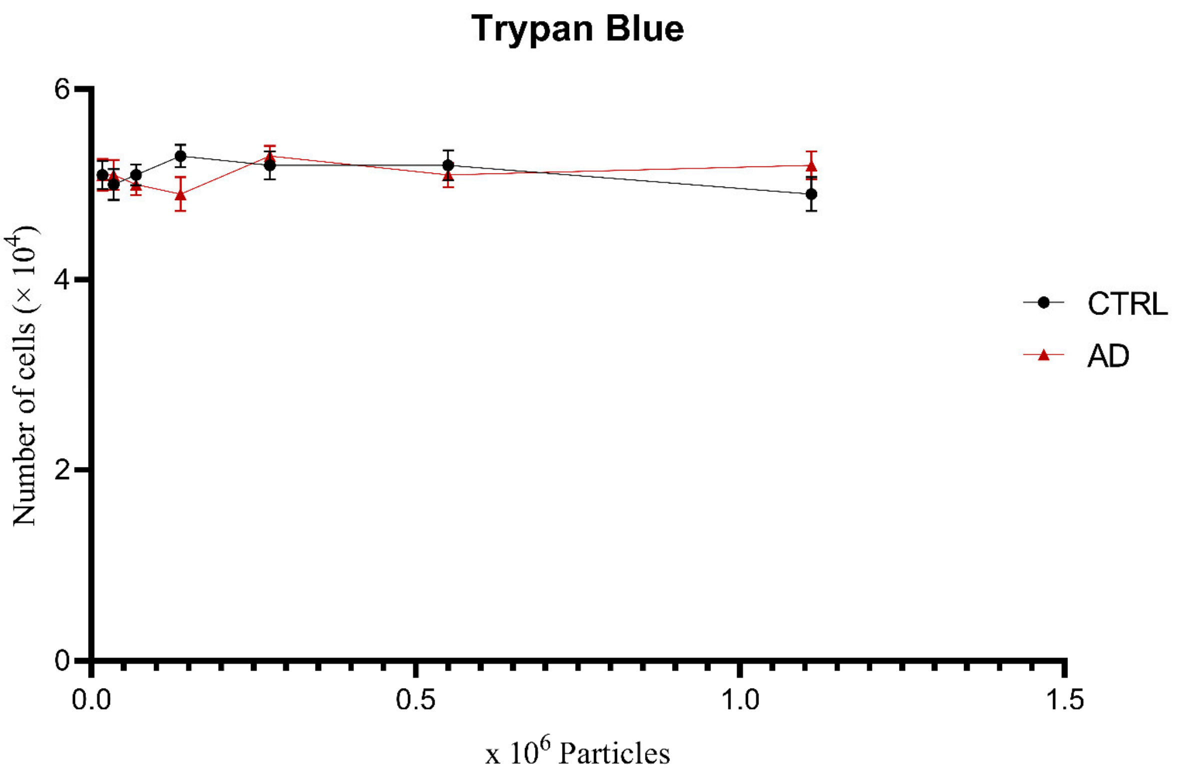

Figure S3. Trypan Blue exclusion assay on CTRL and AD cells treated with the same concentrations of EVs tested with MTT assay, in order to evaluate the potential cytotoxicity. Data are expressed as mean ± SD of three replicates.
